# Polyclonal Multidrug ESBL-Producing *Klebsiella pneumoniae* and Emergence of Susceptible Hypervirulent *Klebsiella pneumoniae* ST23 Isolates in Mozambique

**DOI:** 10.3390/antibiotics12091439

**Published:** 2023-09-12

**Authors:** José João Sumbana, Antonella Santona, Nader Abdelmalek, Maura Fiamma, Massimo Deligios, Alice Manjate, Jahit Sacarlal, Salvatore Rubino, Bianca Paglietti

**Affiliations:** 1 Department of Biomedical Sciences, University of Sassari, 07100 Sassari, Italy; sumbanajj@gmail.com (J.J.S.); asantona@uniss.it (A.S.);; 2 Department of Microbiology, Faculty of Medicine, Eduardo Mondlane University, Maputo P.O. Box 257, Mozambique; 3 Clinical-Chemical Analysis and Microbiology Laboratory, San Francesco Hospital, 08100 Nuoro, Italy

**Keywords:** *K. pneumoniae*, multidrug resistant, ESBL, CTX-M-15, hypervirulent *K. pneumoniae* ST23, Mozambique

## Abstract

Globally, antibiotic-resistant *Klebsiella* spp. cause healthcare-associated infections with high mortality rates, and the rise of hypervirulent *Klebsiella pneumoniae* (hv*Kp*) poses a significant threat to human health linked to community-acquired infections and increasing non-susceptibility. We investigated the phenotypic and genetic features of 36 *Klebsiella* isolates recovered from invasive infections at Hospital Central of Maputo in Mozambique during one year. The majority of the isolates displayed multidrug resistance (MDR) (29/36) to cephalosporins, gentamicin, ciprofloxacin, and trimethoprim–sulfamethoxazole but retained susceptibility to amikacin, carbapenems, and colistin. Most isolates were ESBLs-producing (28/36), predominantly carrying the *bla*_CTX-M-15_ and other beta-lactamase genes (*bla*_SHV_, *bla*_TEM-1_, and *bla*_OXA-1_). Among the 16 genomes sequenced, multiple resistance genes from different antibiotic classes were identified, with *bla*_CTX-M-15_, mostly in the IS*Ecp1*-*bla*_CTX-M-15_-*orf477* genetic environment, co-existing with *bla*_TEM-1_ and *aac(3)-IIa* in five isolates. Our results highlight the presence of polyclonal MDR ESBL-producing *K. pneumoniae* from eight sequence types (ST), mostly harbouring distinct yersiniabactin within the conjugative integrative element (ICE). Further, we identified susceptible hv*Kp* ST23, O1-K1-type isolates carrying yersiniabactin (*ybt1*/ICEKp10), colibactin, salmochelin, aerobactin, and hypermucoid locus (*rmpADC*), associated with severe infections in humans. These findings are worrying and underline the importance of implementing surveillance strategies to avoid the risk of the emergence of the most threatening MDR hv*Kp*.

## 1. Introduction

Several species of *Klebsiella*, including *Klebsiella pneumoniae* (*Kp*) and *Klebsiella oxytoca*, are known as opportunistic pathogens that naturally reside in the nasal passages, throat, skin, and intestinal tract of healthy individuals. However, they can also be responsible for a wide spectrum of infections, such as urinary tract, intra-abdominal, and bloodstream infections, pneumonia, and meningitis [1,2].

Alarmingly, *Klebsiella* have emerged worldwide as multidrug-resistant (MDR) pathogens, driven by an excessive scale of use and misuse of antibiotics, which enhance the development and spread of antimicrobial resistance genes and MDR strains.

Infections caused by these strains, particularly those producing extended-spectrum beta-lactamases (ESBLs) that confer resistance to third-generation cephalosporins and monobactams, and/or carbapenemases resulting in resistance against all β-lactams, including carbapenems, present significant challenges in terms of treatment [3,4]. Such infections can lead to severe illness or even fatalities, especially in individuals with weakened immune systems. They represent a significant global public health concern and are among the leading causes of hospital-acquired infections [5].

Of particular concern is *Kp*’s contribution to the dissemination and evolution of antimicrobial resistance between *Klebsiella* species and other *Enterobacterales*. This can occur through various mechanisms, with plasmids and mobile genetic elements playing a central role [6,7]. Consequently, the implementation of appropriate infection control measures in healthcare settings becomes crucial in order to effectively curb the emergence of antimicrobial resistance resulting from *Kp* infections.

In addition to the classical *Kp* strains, a hypervirulent variant, *Klebsiella pneumoniae* (hv*Kp*), has emerged. These hv*Kp* strains are associated with community-acquired infections and are characterized by the ability to cause severe and rapidly progressive infections, even in healthy individuals [8,9].

They possess a variety of specific virulence factors, including the salmochelin siderophore system (*iroBCDN*), aerobactin cluster (*iucABCD*-*iutA*), genotoxin colibactin (*clB*), and mucoid phenotype regulator (*rmpA* and *rmpA2*) [10], increasing bacterial fitness and pathogenicity. Notably, some lineages produce high levels of polysaccharide K capsule to evade the immune system, especially hv*Kp* isolates from Clonal Group CG23, with sequence type (ST) 23 being dominant [11].

Although hv*Kp* strains are usually susceptible to most classes of antimicrobial agents [10], the merging of virulence and resistance determinants in hybrid lineages has been increasingly reported worldwide, further worsening the situation [12].

Mozambique is facing, as are many other countries, its share of challenges concerning *Klebsiella* infections. Reports of MDR ESBL-producing *Kp* have surfaced [13,14,15,16,17,18], and there have been documented cases of MDR ESBL-producing hv*Kp* isolates causing severe and fatal disease, particularly in vulnerable populations, like children [19].

However, in Mozambique, the available data from the main Maputo hospitals remain limited, and molecular typing of *Klebsiella* isolates is rare in the country, making critical the differentiation among *Klebsiella* isolates for effective infection control allocation. Sequencing technologies have proven valuable in achieving this goal, as well as identifying molecular mechanisms of antibiotic resistance, virulence, and plasmid diffusion.

Due to the clinical significance and knowledge gaps in Mozambique, in this study, we characterized *Klebsiella* spp. isolated from blood, pus, and cerebrospinal fluid over the course of a year to provide an overview of the isolates circulating at Hospital Central of Maputo (HCM) in Mozambique, a national health unit of a quaternary level. Our analysis of the genetic features of *Klebsiella* isolates by polymerase chain reaction (PCR) and whole genome sequencing (WGS) has yielded valuable information that can contribute to the improvement of treatment and infection prevention and control measures.

## 2. Results

### 2.1. Phenotypic and Genotypic Characterization of Klebsiella Isolates

#### 2.1.1. *Klebsiella* Isolates and Antimicrobial Susceptibility Testing

Over a year’s study, a total of 36 non-duplicate *Klebsiella* isolates were identified, of which 35 were *Kp* and 1 was *K. oxytoca*. These isolates were mainly obtained from patient samples in the Paediatrics ward (*n* = 11) and Medicine ward (*n* = 10), with the remaining (*n* = 19) originating from unspecified wards at Hospital Central of Maputo, Mozambique (Table S1b). The majority of these isolates were sourced from blood samples (*n* = 27), followed by seven from pus samples and two from cerebrospinal fluid (CSF).

**Figure 1:**
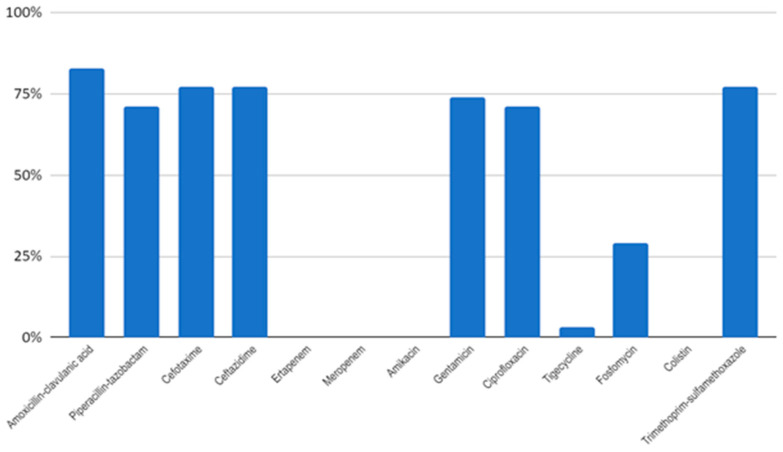
Antimicrobial resistance rates of *K. pneumoniae* isolated in Hospital Central of Maputo, Mozambique.

Among the *Kp* isolates, a significant portion (77%, 27/35) were found to be ESBL producers. Moreover, a substantial number (80%, 28/35) of the isolates exhibited multidrug resistance. The primary resistances observed were against amoxicillin–clavulanic acid (82.8%, 29/35), cefotaxime, ceftazidime, trimethoprim–sulfamethoxazole (77%, 27/35), gentamicin (74.3%, 26/35), piperacillin–tazobactam, and ciprofloxacin (71.4%, 25/35), as well as fosfomycin (28.5%, 10/35). One isolate was also resistant to tigecycline. However, all *Kp* isolates remained susceptible to carbapenems, colistin, and amikacin (Figure 1, Table S1a).

The *K. oxytoca* isolate (SSM90) was an ESBL producer and displayed resistance to amoxicillin–clavulanic acid, cefotaxime, ceftazidime, gentamicin, fosfomycin, and trimethoprim–sulfamethoxazole (Table S1a).

#### 2.1.2. Antimicrobial Resistance Determinants of *Klebsiella* Isolates

In the analysis of beta-lactam resistance determinants, PCR was conducted on all 36 *Klebsiella* isolates. Among the ESBL-producing *Kp* and K. *oxytoca* isolates, 75% (27/36) were found to carry CTX-M-type ESBLs, with *bla*_CTX-M-15_ being the most prevalent variant, present in 22 isolates. Strains carrying *bla*_CTX-M-15_ were highly resistant to antibiotics, including all beta-lactams tested except carbapenems. Additionally, SHV-type beta-lactamases were detected in 78% (28/36) of the isolates, and other TEM β-lactamases were found in 67% (23/36) of them. Notably, no carbapenemase genes or AmpC genes were detected.

Furthermore, a subset of sixteen representative *Klebsiella* isolates was selected for whole genome sequencing throughout the study period, based on their phenotype of antimicrobial resistance, source of isolation, and ward affiliation (Table 1).

The genomic analysis of *Klebsiella* isolates confirmed the presence of *bla*_CTX-M-15_ in 13 of them. Other genes encoding beta-lactamases and oxacillinases were also prevalent: *bla*_TEM-1_ was found in 60% (9/15) of the isolates, while *bla*_SHV_ class-A β-lactamase was detected in 67% (10/15) of the isolates, with both intrinsic variants (*bla*_SHV-1_ and *bla*_SHV-11_) and ESBL variants (*bla*_SHV-2_ and *bla*_SHV-187_) observed. Additionally, *bla*_OXA-1_ was identified in 47% (7/15) of the isolates. Furthermore, in *K. oxytoca*, the presence of *bla*_CTX-M-15_ was associated with the coexistence of ESBL *bla*_OXY-4-1_ and the beta-lactamases *bla*_TEM-1_ and *bla*_OXA-1_ (Table 2).

Regarding the genetic environment of the *bla*_CTX-M-15_ gene, it was observed that in most *Kp* isolates and in *K. oxytoca* SSM90, the IS*ecp1* insertion sequence from the IS*1380* family was located upstream of the *bla*_CTX-M-15_ gene, forming the transposition unit IS*ecp1*-*bla*_CTX-M-15_ -*orf477* (Table 2). However, in *Kp* isolates SSM79 and SSM85, the upstream IS*Ecp1* copy of the *bla*_CTX-M-15_ gene was truncated and replaced with the IS*26* copy (Table 2). According to the prediction of Mlplasmids, IS*ecp1* transposition units were situated on plasmid contigs in all ESBL-carrying isolates, with the exception of isolate SSM73, in which IS*ecp1*-*bla*_CTX-M-15_ -*orf477* was located on a chromosomal contig size of 43,172 bp.

The isolates harboured a diverse array of antimicrobial resistance genes conferring resistance to various classes of antibiotics. Genes associated with resistance to aminoglycosides (*aac(6′)-Ib-cr*, *aac(3)-IIa, aac(3)-IId*, *aac(3)-IIe*, *aph(3″)-Ib*, *aph(6)-Id*, *aadA1*, *aadA2*, *aadA14,* and *aadA16*), quinolones (*qnrB1*, *qnrB6,* and *aac(6′)-Ib-cr*), phenicols (*catA1*, *catA2,* and *catB3*), trimethoprim (*dfrA5*, *dfrA7*, *dfrA12*, *dfrA14,* and *dfrA27*), sulfonamides (*sul1* and *sul2*), macrolide *mph(A)*, tetracycline (*tet(A)* and *tet(D*)), fosfomycin (*fosA*), and rifampicin (*ARR-3*) were also detected (Table 2).

In some instances, antimicrobial resistance genes clustered together within the isolates. For instance, in five isolates, *bla*_CTX-M-15_ genes co-existed with *bla*_TEM-1_ and *aac(3)-IIa*. The presence of *bla*_OXA-1_ was consistently linked with *catB3* and *aac(6′)-Ib-cr* in all cases. Moreover, four isolates were found to possess the combination of *aadA16-dfrA7-arr3-aac(6′)Ib-cr* genes together.

All *Kp* isolates possessed the olaquindox/quinolone AB (*oqxAB*) efflux pump genes mediating low to intermediate quinolone resistance. Furthermore, through ResFinder analysis, point mutations were identified in the porin genes *ompK36* and *ompK37*, several of which were predicted to contribute to the resistance to cephalosporins and favour the selection of carbapenems resistance in ESBL-producing isolates (Table S1b). Similarly, mutations in the efflux pump regulator gene *acrR* were identified in all isolates, except *K. oxytoca* (Table S1b), and were predicted to contribute to heightened resistance to fluoroquinolone.

In addition, the tigecycline-resistant isolate (SSM58P) exhibited a premature stop codon in RamR (E78*), a repressor of RamA that acts as a positive regulator for the AcrAB efflux pump system. This genetic alteration in RamR may potentially contribute to the observed phenotypic resistance to tigecycline [20]. However, all other isolates exhibited a RamR (K194*) stop codon despite having a tigecycline MIC under the EUCAST breakpoint.

The *K. oxytoca* isolate resistant to fosfomycin lacked the *fosA* gene, but it exhibited mutations compared to the fosfomycin-susceptible *K. pneumoniae* K68 strain in the antibiotic target and transporters (MurA, GlpT, and UhpT), including MurA/S148N_S210T, GlpT/I260_VI429L, and UhpT/V434I, which are known to confer resistance to fosfomycin in *Kp* [21].

There were a few discrepancies between the genotype and the phenotype for some antimicrobials: the *Kp* isolate (SSM52A), although it harboured *aac(3)-IIa/aac(6′)-Ib-cr*, was phenotypically susceptible to aminoglycoside, and ten *Kp* isolates phenotypically susceptible to fosfomycin contained a chromosomal *fosA5* gene, suggesting that this gene could be not expressed but may increase the risk of developing resistance during treatment [22].

#### 2.1.3. MLST and Virulence-Associated Genes Analysis of *Klebsiella* Isolates

In silico MLST analysis of the sequenced strains revealed that *Kp* is largely polyclonal, with 10 different sequence types. ST607 (*n* = 4), ST831 (*n* = 2), and ST23 (*n* = 2) were the most represented, and ST13, ST14, ST17, ST394, ST48, ST711, and ST985 were distributed among the isolates (Table 1 and Table 2). The genomic analysis identified virulence factors related to bacterial adhesion and biofilm formation type-1 and type-3 fimbriae *(mrkABCDF),* capsular K locus (KL) (K-type), lipopolysaccharide O locus (O-type), and iron acquisition system *(fyuA, irp and ybt).* Nine different capsular K-types were identified (K1, K2, K3, K25, K18, K39, K54, and K62) (Table S1c).

The O-type 1 was identified in 80% (12/15) of the *Kp* isolates, and four unique O-type variants were identified in the isolates: O2, O5, O2afg, and 1. Iron acquisition genes were found in all but two *Kp* isolates (SSM58P from pus and SSM63 from blood) harbouring a complete yersiniabactin siderophore system apart from one isolate (SSM37) (Table S1c). Five distinct *ybt: ybt9/*conjugative integrative element (ICE) ICE*Kp3 (n* = 4*), ybt10/*ICE*Kp4* (*n* = 1), *ybt14/*ICE*Kp5 (n* = 5, of which 1 was incomplete), and *ybt16/*ICE*Kp12* (*n* = 1) were identified (Table S1c).

Of the two ST23 *Kp* strains, one originated from the bloodstream of a patient in the Medicine ward, while the other originated from the CSF of a paediatric patient. These isolates were associated with K1 capsular (*wzi1*) locus and O1/O2v2 locus and harboured the most extensive array of virulence genes (virulence score 5) considered characteristic biomarkers of hypervirulent variants. These include yersiniabactin (*ybt1/*ICE*Kp10*), colibactin (*clb2*), salmochelin (*iro1*), and aerobactin (*iuc1*). Additionally, ST23 isolates exhibited the regulator of the mucoid phenotype *rmpADC* (lineage 1), and one isolate carried a truncated *rmpA2* locus (Table S1c).

#### 2.1.4. Plasmid Content Analysis

The IncFII(K) replicon plasmid was identified in 77% of the *Kp* isolates (10/15) and was associated with most of the STs. These *Kp* also harboured other plasmids, mainly of the IncFIa, IncFIb, IncR, and IncH groups (Table 2). Five different pMLST IncF were detected, with five belonging to [K5:A-:B-], four to [K13:A13:B-], and one to [K7:A13:B-], [K8:A21:B-], and [K9:A-:B-]. IncHI2 plasmid was found in *K. oxytoca* (SSM90). ST23 hv*Kp* isolates carried *repB* and IncHI1B replicons.

## 3. Discussion

*Klebsiella* is a significant contributor to both community-acquired and hospital-acquired infections, with an alarming rise in antimicrobial resistance. Surveillance of *Klebsiella* antibiotic resistance is therefore mandatory in order to adapt the antibiotic combination to local resistance patterns.

In our study, we focused on determining the phenotypic and genetic features of *Klebsiella* isolated from a quaternary care hospital in Maputo City, the capital of Mozambique, over a period of one year.

Our investigation has revealed worrisome findings concerning *Klebsiella* isolates from extraintestinal sites (mainly blood samples). The majority of these isolates were found to be ESBL-producing and exhibited multidrug resistance to several crucial antibiotics, which were, primarily, trimethoprim–sulfamethoxazole, amoxicillin/clavulanate, cephalosporins, gentamicin, and ciprofloxacin, which are commonly used to treat severe *Enterobacteriaceae* infections prevalent among the children in the region.

These findings raise concerns regarding the sustainability of these drugs’ efficacy in treating those infections in Mozambique, forcing the use of second-line antibiotics. *Klebsiella* isolates from this study were susceptible to carbapenems and colistin, which are usually considered last-resort therapeutic options for treating infections when other antibiotics fail. However, these alternative options are frequently unaffordable or unavailable in the country, creating significant challenges in managing infections effectively [14].

In our study, the prevalence of ESBL-producing *Klebsiella* isolates is alarming due to the high rate of beta-lactamases, including CTX-M-15 and other types, such as TEM, OXA, and SHV. ESBL-producing *Klebsiella* isolates are spreading worldwide [23], including in Africa [24,25]. In particular, in Mozambique, CTX-M-15 has been often reported in *E. coli* [26,27,28], in *Klebsiella* [14,16], and, recently, also in *Enterobacter cloacae* complex isolates [29]. In our studied isolates, the dissemination of the *bla*_CTX-M-15_ gene could be occurring through the mobility of plasmids, with IncF being the most common type, and the mobilization element IS*Ecp1* or IS26 truncating IS*Ecp1,* which have been associated with the horizontal dissemination of resistance to beta-lactams [30]. Interestingly, we have also noticed the same genetic context around *bla*_CTX-M-15_ (e.g._,_ IS*Ecp1*-*bla*_CTX-M-15_-*orf477*), which was also observed in extraintestinal *E. coli* strains from the same hospital [27], suggesting a possible interspecies intra-hospital spread.

Additionally, in the SSM73 *Kp* isolate, we observed the integration of CTX-M-15 into the chromosome via IS*Ecp1*. The placement of resistance traits on chromosomes has elevated the worrying scenario to a new level, allowing these clones to stably spread within clinical settings, regardless of any environmental pressures [31]. We have also observed an association between the carriage of CTX-M-15, *bla*_TEM-1_, and *aac(3)-IIa* genes within isolates, suggesting possible co-selection mechanisms, where the acquisition of one resistance gene may facilitate the spread of others due to the selective pressures imposed by antibiotic use.

Moreover, these isolates harboured numerous resistance genes against various antibiotic classes. This accumulation of resistance underscores the complexity of antimicrobial resistance profiles in these *Klebsiella* isolates, posing a significant threat to public health. Immediate attention is required to address this issue before it becomes even more challenging to manage in the future.

As expected, WGS data verified that the carbapenem-susceptible isolates did not contain any carbapenemase genes. Nevertheless, certain alterations, such as I70M, I128M, and N230G, were found in the porin OmpK37. Although these alterations were not accompanied by a reduction in carbapenem MIC, they may enable antibiotics transportation into bacterial cells, thereby potentially giving rise to carbapenem resistance [32]. Therefore, the administration of carbapenems in this region should be approached with caution. Moreover, carbapenem resistance mediated by NDM in *E. coli* has been already documented in this hospital [28], which heightens the risk of *Klebsiella* isolates acquiring carbapenem resistance through NDM mechanisms. This situation emphasizes the importance of vigilant surveillance and infection control measures to address the potential spread of carbapenem resistance in clinical settings.

The typing analysis by MLST highlighted the circulation of multiple STs (ST13, ST14, ST17, ST23, ST48, ST394, ST607, ST711, ST831, and ST985) among *Kp* isolates at HCM, with ST14, ST48, and ST23 having a global distribution. This diversity in sequence types is in line with findings from other reports [24,25], although none of them belong to the most common epidemic clones associated with antimicrobial resistance as ST258 [23] (which is, however, relatively rare in Africa) [24]. ST607 was the most represented sequence type in our study, and it has been commonly identified in *Kp* isolates carrying *bla*_CTX-M-15_ from Malawi, South Africa, and Tanzania, neighbouring countries of Mozambique [24,25,33,34], showing the possibility of clone transmission between these countries.

Interestingly, the yersiniabactin siderophore system was found in the majority of the MDR *Kp* isolates, including ST14 and ST48. In particular, acquiring yersiniabactin greatly affects the severity of *Kp* infection and has been linked to an increased risk of bacteraemia and sepsis in these lineages. Additionally, it seems that obtaining yersiniabactin is often the first step in acquiring additional siderophores, which enhances their ability to cause invasive infections within communities [23].

It is concerning that ST23 O1-K1-type hv*Kp* isolates carrying multiple virulence genes have been identified for the first time in Mozambique, as these strains are rarely encountered in Africa, with limited reports from Algeria, South Africa, and Madagascar [11,35,36]. The identification of hv*Kp* strains is significant in a clinical context, as they have the potential to cause infections that necessitate prolonged antibiotic treatment and are prone to disseminate through the body. Thankfully, these hv*Kp* are currently responsive to the majority of antibiotics.

However, as seen for MDR *Kp* clones, hypervirulent ST23 isolates also displayed several mutations in Omp36/37 and AcrR proteins, which underlines their potential contribution to developing resistance to cephalosporins, carbapenems, and fluoroquinolones during treatment.

Furthermore, the presence of MDR ESBL-producing *Klebsiella* isolates carrying *rmpA* and *wzi*-K1 genes associated with hypermucoviscosity has been previously documented at the Manhiça District Hospital [19], suggesting the need to monitor the spread of these potentially dangerous clones locally.

In Mozambique, the combination of insufficient resources for infection control and improper use of antimicrobial therapy has led to a significant issue with antibiotic resistance. The results of this investigation provide valuable insights into the genotypic and phenotypic attributes displayed by Mozambican *Klebsiella* isolates. In particular, the presence of MDR ESBL-producing *Klebsiella* spp. and ST23 hv*Kp* isolates raises concerns regarding the emergence of highly-drug-resistant clones that have access to mobile pools of virulence and antimicrobial resistance genes. This scenario could significantly hamper effective infection management efforts in hospitals and limit available treatment strategies, making it imperative to take immediate action to address this critical problem.

## 4. Materials and Methods

### 4.1. Sample Collection and Bacterial Identification

Gram-negative bacteria routinely isolated from blood, pus, and cerebrospinal fluid (CSF) samples obtained from patients admitted at the Hospital Central of Maputo (HCM) between March 2017 and July 2018 were collected. This study was conducted with ethical approval provided by the National Health Bioethics Committee of Mozambique (Ref. 78/CNBS/2017).

The isolation of suspected *Klebsiella* was carried out at the Microbiology Laboratory of HCM. Sheep blood, chocolate, MacConkey, and XLD agar plates were utilized to culture and isolate the bacteria. Preliminary identification was performed using biochemical methods at the Microbiology Laboratory in the Faculty of Medicine of Eduardo Mondlane University in Maputo, Mozambique. These isolates were later sent to the Department of Biomedical Sciences at the University of Sassari in Italy for species-level identification using matrix-assisted laser desorption/ionisation time-of-flight mass spectrometry (MALDI-TOF/MS) with a MALDI Biotyper instrument (Bruker Daltonics Inc., Billerica, MA, USA) and for further investigations, including antibiotic susceptibility testing as well as whole genome sequencing (WGS) and subsequent analysis.

### 4.2. Antibiotic Susceptibility Testing

Antibiotic susceptibility testing was undertaken by the Vitek2 system, with a GN377-specific card for Gram-negative bacteria (bioMérieux, Marcy-l’Étoile, France). In total, 13 antimicrobials agents were tested, including amoxicillin–clavulanic acid (AMC), piperacillin–tazobactam (TZP), cefotaxime (CTX), ceftazidime (CAZ), ertapenem (ETP), meropenem (MEM), amikacin (AMK), gentamicin (GEN), ciprofloxacin (CIP), tigecycline (TGC), fosfomycin (FOF), colistin (CST) and trimethoprim–sulfamethoxazole (SXT), following European Committee on Antimicrobial Susceptibility Testing (EUCAST) 2018 interpretative criteria. Isolates resistant to one agent of ≥3 classes were considered MDR. ESBL production was performed by phenotypic confirmatory disc diffusion test using the ESBL disc kit (Liofilchem, Teramo, Italy).

### 4.3. PCR Detection of β-Lactamase Genes

All 36 *Klebsiella* isolates were preliminarily screened by PCR amplification using genome DNA extracted from the overnight nutrient broth cultures using a Wizard^®^ Genomic DNA Purification Kit (Promega, Madison, WI, USA) following the manufacturer’s instructions. Specific primers for several resistance genes encoding for narrow-spectrum beta-lactamases and ESBLs (*bla*_TEM_, *bla*_SHV_, *bla*_CTX-M_, *bla*_CTX-M-2,_ *bla*_CTX-M-9,_ *bla*_CTX-M-15_, *bla*_GES_, *bla*_VEB_, and *bla*_PER_), AmpCs (MOXM, CITM, DHAM, ACC, EBCM, and FOXM), carbapenemases (KPC, OXA-48-like, IMP, VIM, and NDM) and PCR conditions were used following previously published protocols [37,38,39,40].

### 4.4. Whole Genome Sequencing (WGS) and Analysis

For WGS, a total of 16 *Klebsiella* isolates originating from different wards were selected for whole genome sequencing throughout the study period, based on their phenotype of antimicrobial resistance and source of isolation. Among these, seven were recovered from pediatric patients’ samples, mostly from blood, and one from cerebrospinal fluid. Additionally, five isolates were obtained from the Medicine ward, and four isolates originated from unspecified wards. Most of the sequenced isolates were multidrug resistant and ESBL producers.

Genome DNAs were extracted as above and, after quantification by Qubit 4 Fluorometer (ThermoFisher, MA, USA), they were sequenced by the Illumina NextSeq platform, at a 30× coverage at NGX Bio (San Francisco, CA, USA). The generated short reads were de novo assembled using the SPAdes 3.13.0. web-based tool (http://cab.spbu.ru/software/spades/, accessed on 10 April 2019). Sequences underwent quality control with FastQC and were deposited in the NCBI database under Bioproject accession number PRJNA951736. The assembled genomes of *Klebsiella* isolates were subjected to analysis with different open online resources. The MLST 2.0 database was used to determine the multilocus sequence type (ST), hosted by the CGE online, available at http://www.genomicepidemiology.org (accessed on 22 March 2023). ResFinder 4.1 (https://cge.cbs.dtu.dk/services/ResFinder/, accessed on 22 March 2023) and the NCBI National Database of Antibiotic Resistant Organisms (NDARO) were used to identify acquired antimicrobial resistant genes and chromosomal mutations. PlasmidFinder 2.1 (https://cge.food.dtu.dk/services/PlasmidFinder/, accessed on 22 March 2023) and pMLST 2.0 (https://cge.food.dtu.dk/services/pMLST/, accessed on 22 March 2023) were utilized for the identification and typing of plasmid replicons. The *Kleborate* [41] bioinformatic tool (https://github.com/katholt/Kleborate) (accessed on 25 March 2023) was used for the presence of the siderophore virulence loci *ybt* (yersiniabactin), *iro* (salmochelin), and *iuc* (aerobactin), the genotoxin locus *clb* (colibactin), and the hypermucoidy genes *rmpA* and *rmpA2*. *Kleborate* also provides a virulence score ranging from 0 to 5: 0 = negative for all of *ybt*, *clb*, and *iuc*; 1 = *ybt* only; 2 = *ybt* and *clb* or *clb* only; 3 = *iuc* only; 4 = *iuc* and *ybt*; and 5 = *ybt*, *clb,* and *iuc*. *Kaptive* Web [42] (https://kaptive-web.erc.monash.edu/) was used to identify capsule polysaccharide (K-type) and lipopolysaccharide (O-type) serotypes (accessed on 22 March 2023). *Mlplasmids* [43] (https://sarredondo.shinyapps.io/mlplasmids) was used to predict if contigs were either plasmid-derived or chromosome-derived, by using *K. pneumoniae* as the species model (accessed on 31 March 2023).

## Figures and Tables

**Table 1:** Characteristics, antimicrobial resistance profiles, and genotyping of *K. pneumoniae* and *K. oxytoca* isolates from Central Hospital of Maputo, Mozambique.

Isolates	Ward	Source	ST	Resistant Profile	*oqxA/B*	*qnrB1*	*qnrB6*	*aac(6′)-Ib-cr*	*aac(3)-IIa*	*aac(3)-IId*	*aac(3)-IIe*	*aph(3″)-Ib*	*aph(6)-Id*	*aadA1*	*aadA2*	*aadA14*	*aadA16*	*TEM-1*	*SHV*	*CTX-M-15*	*OXA-1*	*OXY-4-1*	*catA1*	*catA2*	*catB3*	*drA5*	*dfrA7*	*dfrA12*	*dfrA14*	*dfrA27*	*sul1*	*sul2*	*mph(A)*	*tet(A)*	*tet(D)*	*fosA*	*ARR-3*
SSM90 ^a^	P	B	NC	AMC-CTX-CAZ-GEN-FOF-SXT																																	
SSM35	P	B	ST13	AMC-TZP-CTX-CAZ-GEN-CIP-FOF-SXT																																	
SSM79	P	B	ST14	AMC-TZP-CTX-CAZ-GEN-CIP-SXT																																	
SSM63	P	B	ST17	AMC-FOF																																	
SSM25L	P	C	ST23	FOF																																	
SSM56	M	B	ST23	FOF																																	
SSM58P	M	P	ST394	AMC-TZP-CTX-CAZ-GEN-CIP-TGC																																	
SSM85	M	B	ST48	AMC-TZP-CTX-CAZ-GEN-CIP-SXT																																	
SSM37	na	B	ST607	AMC-TZP-CTX-CAZ-GEN-CIP-SXT																																	
SSM56P	M	P	ST607	AMC-TZP-CTX-CAZ-GEN-CIP-SXT																																	
SSM64	P	B	ST607	AMC-TZP-CTX-CAZ-GEN-CIP-SXT																																	
SSM114	P	B	ST607	AMC-TZP-CTX-CAZ-GEN-CIP-SXT																																	
SSM73	M	B	ST711	AMC-TZP-CTX-CAZ-GEN-CIP-FOF-SXT																																	
SSM52A	na	B	ST831	AMC-CTX-CAZ-CIP-SXT																																	
SSM52B	na	B	ST831	AMC-TZP-CTX-CAZ-GEN-SXT																																	
SSM10P	na	P	ST985	AMC-TZP-CTX-CAZ-GEN-CIP-SXT																																	

^a^ *Klebsiella oxytoca*. Abbreviations: Ward: P, Paediatric; M, Medicine; Source: B, blood; P, pus; C, cerebrospinal fluid; ST, Sequence type; NC, not classifiable; na, not available. Antimicrobials: AMC, amoxicillin–clavulanic acid; TZP, piperacillin–tazobactam; CTX, cefotaxime; CAZ, ceftazidime; ETP, ertapenem; MEM, meropenem; AMK, amikacin; GEN, gentamicin; CIP, ciprofloxacin; TGC, tigecycline; FOF, fosfomycin; CST, colistin; SXT, trimethoprim–sulfamethoxazole. ESBLs and other beta-lactamase genes are shown in green squares, other antimicrobial resistance genes are shown in red squares.

**Table 2:** Beta-lactamases, genetic environment of CTX-M-15, and plasmid replicons of *Klebsiella* isolates from Mozambique.

Isolates	ST	Beta-Lactamase Enzymes	Genetic Context of CTX-M-15	Plasmids Replicons	IncF RST
SSM90 ^a^	NC	CTX-M-15, TEM-1B, OXA-1, OXY-4-1 ^b^	IS*ecp1*-*bla*_CTX-M-15_-*orf477*	IncHI2, IncHI2A	-
SSM35	ST13	CTX-M-15, OXA-1, SHV-1	IS*ecp1*-*bla*_CTX-M-15_-*orf477*	IncFII(K), Col, FIB, repB	[K5:A-:B-]
SSM79	ST14	CTX-M-15, TEM-1B, OXA-1, SHV-28	IS*26-*ΔIS*ecp1-bla*_CTX-M-15_*-orf477*	IncFIB(K), Col, FII	[K9:A-:B-]
SSM63	ST17	TEM-1B, SHV-11	-	FIA, FIB, FII	[K8:A21:B-]
SSM25L	ST23	SHV-11	-	repB, HI1B	-
SSM56	ST23	SHV-11	-	repB, HI1B	-
SSM58P	ST394	CTX-M-15, TEM-1B, SHV-11	IS*ecp1*-*bla*_CTX-M-15_-*orf477*	IncFII(K), Col, FIB	[K13:A-:B-]
SSM85	ST48	CTX-M-15, TEM-1B, SHV-1, OXA-1	IS26*-*ΔIS*ecp1*-*bla*_CTX-M-15_-*orf477*	IncFII(K), Col, FIB	[K5:A-:B-]
SSM114	ST607	CTX-M-15, TEM-1B, SHV-1	IS*ecp1*-*bla*_CTX-M-15_-*orf477*	IncFII(K), FIA, FIB, R	[K7:A13:B-]
SSM37	ST607	CTX-M-15, TEM-1B, SHV-1	IS*ecp1*-*bla*_CTX-M-15_-*orf477*	IncFII(K), FIA, FIB, R	[K13:A13:B-]
SSM56P	ST607	CTX-M-15, TEM-1B, SHV-1	IS*ecp1*-*bla*_CTX-M-15_-*orf477*	IncFII(K), FIA, FIB, R	[K13:A13:B-]
SSM64	ST607	CTX-M-15, TEM-1B, SHV-2 ^b^	IS*ecp1*-*bla*_CTX-M-15_-*orf477*	IncFII(K), FIA, FIB, R	[K13:A13:B-]
SSM73	ST711	CTX-M-15, TEM-1B, OXA-1, SHV187 ^b^	IS*ecp1*-*bla*_CTX-M-15_-*orf477*	Col(BS512)	-
SSM52A	ST831	CTX-M-15, TEM-1B, OXA-1, SHV-11	IS*ecp1*-*bla*_CTX-M-15_-*orf477*	IncFII(K), FIB, HI1B	[K5:A-:B-]
SSM52B	ST831	CTX-M-15, TEM-1B, OXA-1, SHV-11	IS*ecp1*-*bla*_CTX-M-15_-*orf477*	IncFII(K), FIB, HI1B	[K5:A-:B-]
SSM10P	ST985	CTX-M-15, TEM-1C, OXA-1, SHV187 ^b^	IS*ecp1*-*bla*_CTX-M-15_-*orf477*	IncFII(K), FIB, HI1B	[K5:A-:B-]

^a^ *Klebsiella oxytoca*; ^b^ Extended-spectrum beta-lactamases; IncF RST, IncF plasmid replicon sequence typing. *bla*_CTX-M-15_ clustered together with *bla*_TEM-1_ and *aac(3)-IIa* in isolates SSM 37, 56P, 58P, 64, 114; *bla*_OXA-1_ clustered together with *catB3* and *aac(6′)Ib-cr* in isolates SSM 10P, 35, 52A, 52B, 73, 79, 85, 90.

## Data Availability

The data presented in this study are available in the article and in the Supplementary Materials.

## Supplementary Materials

The following supporting information can be downloaded at https://www.mdpi.com/article/10.3390/antibiotics12091439/s1. Table S1a: Date, ward, source, and MIC values (mg/L) of antimicrobial agents for the 36 *Klebsiella* isolates from Hospital Central of Maputo; Table S1b: Mutations of *ompK36/ompK37* and *acrR* associated with different antimicrobial resistance among sequenced *Klebsiella* isolates; Table S1c: Molecular characterization of virulence factors in *Klebsiella* isolates.
